# COVID-19 and the Long-Term Care Ombudsman Program: Findings From a National Study

**DOI:** 10.1093/geroni/igab046.331

**Published:** 2021-12-17

**Authors:** Lori Smetanka, Chenguang Du, Pamela Teaster, Kathryn Ratliff

**Affiliations:** 1 The National Consumer Voice for Quality Long-Term Care, Washington, District of Columbia, United States; 2 Center for Gerontology, Virginia Tech, BLACKSBURG, Virginia, United States; 3 Virginia Tech, BLACKSBURG, West Virginia, United States

## Abstract

The purpose of our study was to explore changes for long-term care ombudsman programs across the country in response to the COVID-19 pandemic. The study team explored the effect of COVID-19 on programs: cases, resident engagement, complaint Investigation and resolution, services, complaints, changes in visitation, and preparedness plans. The research team developed survey items and beta-tested them with state and local LTCOs prior to distributing the survey nationally to State Long-Term Care Ombudsman and Local Long-Term Care Ombudsman in order to characterize experiences of the participants. From 62 state LLTC respondents we learned there were 81.0% fewer cases received, 97.36% were less able to engage with residents, 78.95% were less involved in the engagement of residents in complaint investigation and resolution and there were 71.05% fewer activities involved in investigations. Not surprisingly, there was an 80.0% increase in information that the LTCO provided to the media.

